# Heterogeneity of tumor immune microenvironment in malignant and metastatic change in LUAD is revealed by single-cell RNA sequencing

**DOI:** 10.18632/aging.204752

**Published:** 2023-06-16

**Authors:** Haiqiang Wang, Guoliang Han, Jiakuan Chen

**Affiliations:** 1 Department of Thoracic Surgery, Tangdu Hospital Air Force Medical University, Fourth Military Medical University, Xian, Shanxi, China

**Keywords:** lung adenocarcinoma, single-cell, immune microenvironment, T regulatory cells, biomarkers

## Abstract

Lung adenocarcinoma (LUAD) is the most common type of non-small cell lung cancer and accounts for approximately 40% of all lung cancer cases. Multiple distant metastases are the major cause of mortality in lung cancer. In this study, single-cell sequencing datasets of LUAD were utilized to depict the transcriptome characteristic of LUAD based on the bioinformatic method. Firstly, the transcriptome landscape of heterogeneous cell types in LUAD was analyzed and memory T cells, NK cells, and helper T cells were revealed to be the common immune cells in tumor, normal, and metastasis tissue, respectively. Then, marker genes were calculated and 709 genes were identified to play a vital role in the microenvironment of LUAD. While macrophages were reported to act as one of the cells in LUAD, enrichment analysis of macrophage marker genes revealed the important role of macrophages in the activation of neutrophils. Next, the results of cell-cell communication analysis suggested that pericytes interact with broad immune cells via MDK-NCL pathways in metastasis samples, MIF-(CD74+CXCR4) and MIF-(CD74+CC44) interaction especially occurred between different cell types in tumor and normal samples. Finally, bulk RNA-seq was integrated to validate the prognosis effect of the marker gene and the maker gene of M2 macrophage, CCL20, showed the most related to LUAD prognosis. Besides, ZNF90 (Helper T cells), FKBP4 (memory T, helper T, Cytotoxic T, and B cells), CD79A (B cells), TPI1 (pericyte), and HOPX (epithelial cells, pericytes) were also pivotal in the pathology of LUAD, helping researchers understand the molecular insight of microenvironment in LUAD.

## INTRODUCTION

Lung adenocarcinoma (LUAD) is the most common histological type of lung cancer and has the highest cancer mortality rate worldwide [1]. Immunotherapies had presented sustained clinical significance in LUAD [2]. However, due to intrinsic genomic and immunogenic heterogeneity, immunotherapy had limited efficacy and the effect for different patients may be inconsistent [3]. To unravel the role of the distinct genetic features of lung cancer subtypes among patients, new research opportunities and technologies have arisen that might contribute to different therapeutic decisions [4, 5]. Plenty of research had reported that the tumor immune microenvironment (TIME) impacts cancer progression and metastases, as well as affects patient prognoses and outcomes [6, 7], which emphasizes the important role of immune cells, vital components of the tumor microenvironment, impacting on patient survival and tumor progression [8, 9]. Although researchers had made great efforts in the past decades, the related information about the role of TIME’s characteristics in LUAD is limited.

Recently, next-generation sequencing, especially single-cell RNA sequencing is a possible option for the analysis of detailed cell population subtypes of LUAD from bulk tissue samples at single cell level [10, 11]. Previous researchers had conducted a comprehensive analysis of single-cell RNA-sequencing data of LUAD and identified prognostic signatures based on B cells [12] and natural killer cells [13]. Then, multivariate analysis was performed to demonstrate that the signature was an independent prognostic factor and highly correlated with immune-cell infiltrations and immune-suppressive states. Although several studies revealed the important role of immune cells and related marker genes impact on LUAD prognosis and process, a comprehensive understanding of TIME malignant and metastatic change in LUAD is still limited. In this study, we collected single-cell sequencing datasets of LUAD and applied an integrated bioinformatic method to depict the transcriptome characteristic of LUAD in normal, tumoral, and metastasis condition. Firstly, the transcriptome landscape of heterogeneous cell types in LUAD was analyzed and marker genes of each cell type were calculated, which were identified to play a vital role in the microenvironment of LUAD. GO and KEGG enrichment analysis, as well as GSEA, of marker genes were performed to explore the functional pathologies. Next, cell-cell communication analysis and trajectory analysis was performed and bulk RNA-seq were integrated to validate the prognosis effect of the marker genes. We aim to broaden the understanding of molecular insight of microenvironment in the malignant and metastatic change in LUAD and the results of the study provide novel insights into the TIME of LUAD patients in different pathological conditions, improve patient prognoses, and provide guidance for selecting clinical LUAD treatments.

## MATERIALS AND METHODS

### Data procession

Single-cell transcriptome files of GSE123902 [14] were downloaded from the Gene Expression Omnibus (GEO) database (http://www.ncbi.nlm.nih.gov/geo/) [15]. Bulk tumor transcriptome profiles and clinical information about LUAD were downloaded from The Cancer Genome Atlas (TCGA) database [16] via the UCSC Xena browser (http://xenabrowser.net/) [17]. R software (version: 4.1) was used for all the analyses in the manuscript.

### Single-cell RNA seq analysis

Two single-cell transcriptome profiles from GSE123902 were collected and the “Seurat” package was applied to perform the single-cell RNA-seq analysis. The batch effect from studies was removed with regularized negative binomial regression by the “Seurat” package. The non-linear dimensional reduction was performed with the t-SNE method. Cluster biomarkers were found by the “Seurat” package.

### Cell-type specific markers identification

Analysis of differentially expressed genes (DEGs) between different samples in certain clusters was performed using the function “FindAllMarkers” in “Seurat” package [18] with standard comparison parameters. Invalid genes were removed and a threshold of absolute log2 fold change > 1 and a significance threshold of adjusted *p*-value < 0.05 were chosen as criteria for valid DEGs. Then, cell-type-specifically expressed genes were also calculated with Seurat, and genes satisfying the above conditions were retained. Finally, take the intersection of DEGs between samples and cell-type-specifically expressed genes to be Cell-type specific marker genes.

### Pathway enrichment analysis

The Gene Set Variation Analysis (GSVA) [19] R package was used for Gene Set Enrichment Analyze (GSEA) [20] of biological processes. The clusterProfiler [21] R package was applied to Gene Ontology (GO) enrichment analysis [22] and Kyoto Encyclopedia of Genes and Genomes (KEGG) enrichment analysis [23]. Genes related to immune-related pathways were collected from the Molecular Signatures Database (MSigDB) [24] and The GSEA algorithm was used to quantify the scores of immune-related pathways to explore the immune-relevant biological processes.

### ssGSEA implementation

The marker genes of 28 immune cell subtypes were collected from public datasets. Infiltration levels of different immune cells, including innate and adaptive immunity cells in each LUAD sample were quantified using single-sample gene-set enrichment analysis (ssGSEA) [25]. We also used the estimation of stromal and immune cells in malignant tumors using expression data (ESTIMATE) algorithm [26] to calculate immune scores and stromal scores to determine levels of TIME immune.

### Survival analysis

The mRNA profiles and related clinical information of LUAD samples were collected from TCGA database. Survival analysis based on the Cox model was used to estimate the survival risk of patients under different conditions. For a specific marker gene, patients were divided into two groups according to median gene expression. Then, Kaplan-Meier curve analysis was performed with the R package “Survival” [27] and the *p*-value between the two groups was also calculated. Marker genes (*p* < 0.05) were identified to be biomarkers significantly related to LUAD prognosis.

### Regulatory element analysis

Important regulatory transcription factors in tumor, normal, and metastasis samples were analyzed by single-cell regulatory network inference and clustering (SCENIC) algorithm [28]. To save calculation time, 1000 cells were randomly selected for analysis, and an important transcription factor was obtained according to the co-expression analysis of TF-target, which interacted with 2592 pairs of TF-targets, and regarded the regulatory network formed by TF and its target gene as a regulatory module, and calculated the area under the curve (AUC) value of the module to characterize its activity. The activity of different regulatory modules in different cell groups and sample types is different, indicating that the function of transcription factors is different in different tissue states.

### Trajectory analysis

Monocle3 [29] was applied to explore differences in the developmental trajectories of immune cells between different samples. Trajectory analysis of different cell types in different samples shows that cell development trajectories are different between different samples, such as B cells have no obvious developmental relationship in the normal sample, while the cells in the tumor sample are divided into 3 clusters, and there are obvious transcript differences between each cluster, that is, differences in developmental relationships.

### Cell-cell communication analysis

Signaling crosstalk is critical for informing diverse cellular decisions, including decisions to activate cell cycle or programmed cell death, undergo migration or differentiate along the lineage [30, 31]. Cell-cell interactions were analyzed using Cellchat [32], an open-source R package (https://github.com/sqjin/CellChat) to infer, visualize and analyze intercellular communications from scRNA-seq data, comparing differences in cell-to-cell interactions between sample classes. The result shows the intercellular interaction between normal samples and metastatic, tumor samples, it revealed that in normal samples, macrophage-related signaling pathways are more active, while in metastasis samples, macrophage-related signaling pathways are missing, and epithelial cells in turn interact more with Regulatory T cells and monocyte.

### Immunocyte abundance calculation

T cells, one of the important components of the immune system, are divided into subpopulations in this project. Including Helper T cell, Memory T cell, Cytotoxic T cell, etc., but immune cells, including B cells and dendritic cells, have more subdivided subsets, it is difficult to distinguish them according to cell marker molecules, so the marker gene of immune cell subsets is collected, and the ssGSEA method is used to calculate the enrichment fraction of T cell subset genes in each cell, and the enrichment score is high. It indicates that the cell has a high functional similarity with the reference T cell.

## RESULTS

### Transcriptome landscape of heterogeneous cell types in lung adenocarcinomas (LUAD)

**Figure 1:**
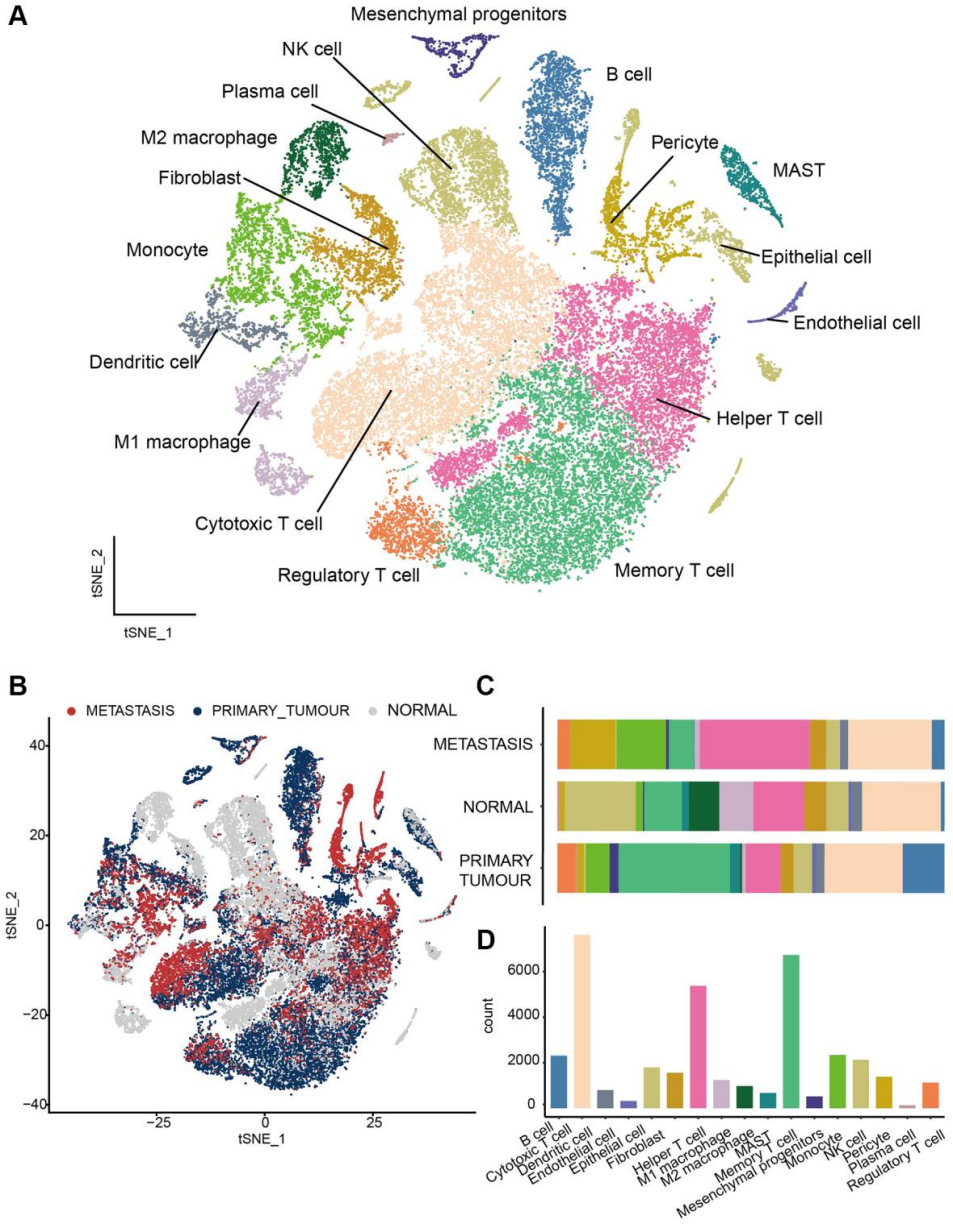
**Transcriptome landscape of heterogeneous cell types in LUAD.** (**A**) All the cells were assigned to 17 cell types. (**B**) The distribution of cell clusters among three groups, including primary tumors, metastasis lesions and normal tissues. (**C**) The fraction of immune cell types in different sample classes. (**D**) Histogram to visual Cell numbers in each cell type.

We collected single-cell sequencing dataset of 17 LUAD donors from GEO database (GSE123902). After strict quality control (see Method), we screened 37030 cells, consisting of 17908 cells from primary tumor samples, 8143 cells from metastasis samples, and 10988 cells from normal samples. To investigate the heterogeneity in different sample classes, all the scRNA-seq data were merged and normalized to create a global cell atlas [14]. Clustering with t-distributed stochastic neighbor embedding (TSNE) algorithm, all the cells were clustered into 28 clusters and then assigned to 17 cell types (Figure 1A; Supplementary Table 1) based on canonical markers reported previously [14, 33–40]. According to the clinical pathology, these samples were divided into primary tumor, metastasis, and normal groups (Figure 1B). Cytotoxic T cells and Memory T cells were the most common cells and immune cells, with 11 subpopulations of B cells, cytotoxic T cells, dendritic cells (DC), Helper T cells, M1 macrophage, M2 macrophage, Mast cells, memory T cells, monocyte, NK cells, and regulatory T cell, come up of 84.58% (31320/37030) of all the cells, thus provide us the opportunity to look insight of the immune microenvironment in different pathological samples.

Next, we elucidated the fraction of immune cell types in different sample classes (Figure 1C). In different pathological conditions, immune cells emerged in different abundance [41]. Based on comparations of normal, metastasis, and tumor tissues of all immune cells, Memory T cell was predominant in tumor tissue, while NK cells were prevalent in normal tissue and Helper T cells were the common immune cells in metastasis tissue. Besides, the proportion of pericytes and monocyte were much higher in metastasis tissue than in tumor and normal tissue and the proportion of B cell was higher in tumor tissue. In contrast, Cytotoxic T cell was the most common immune cells (Figure 1D) while the distribution of cytotoxic T cell between different samples in more even. Taken together, our scRNA-seq analyses dissected the heterogeneity of the immune landscape between normal, tumor, and metastasis samples of LUAD, and different sample classes have different dominant cells.

### Identification of marker genes for types of immune cells in different sample classes

**Figure 2:**
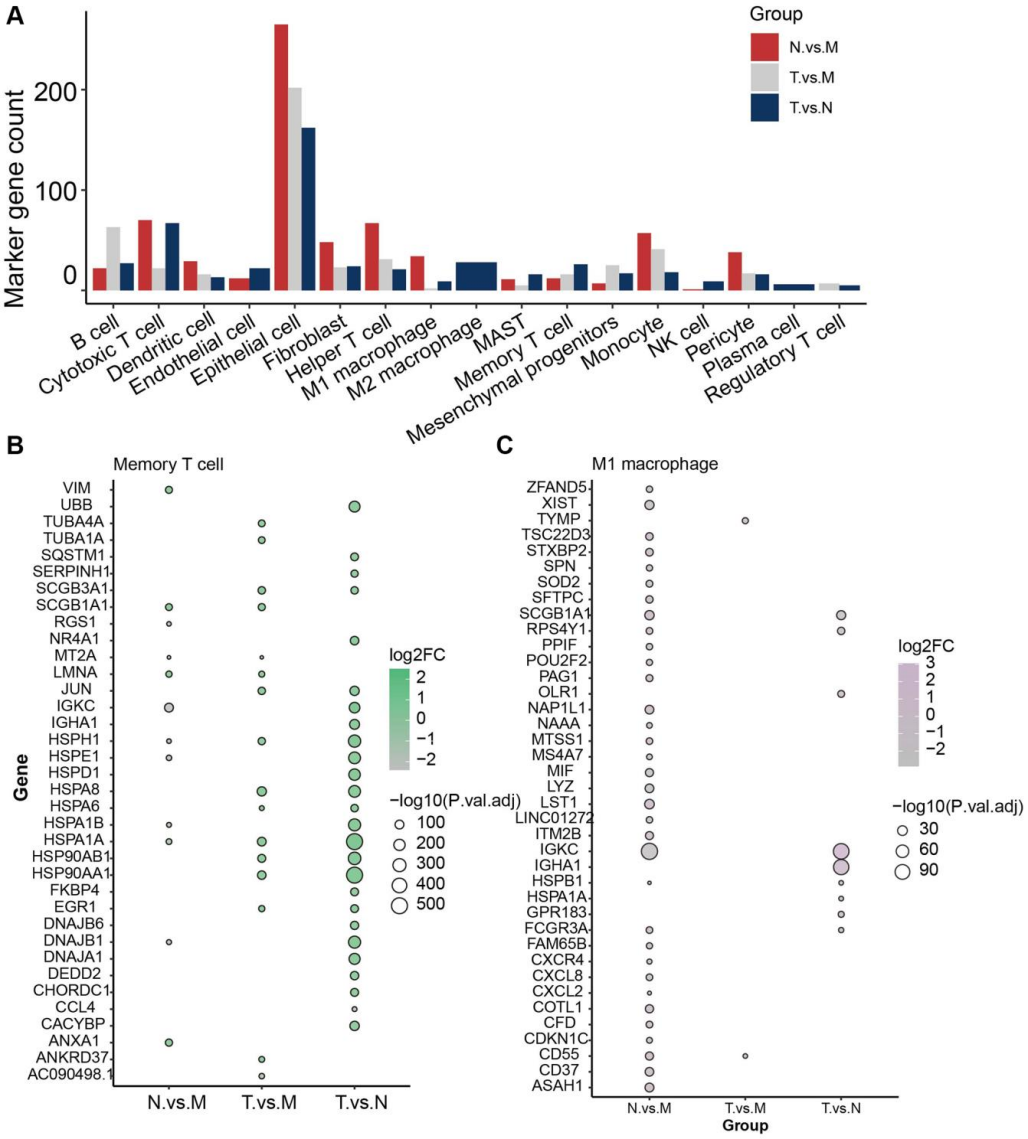
**Identification of marker genes for type of immune cells in different sample classes.** (**A**) Overview of total 709 marker genes. (**B**) Marker genes identified in memory T cells. (**C**) Marker genes identified in M1 macrophages.

To further unveil the role of the immune cells in their microenvironment in different sample classes, we then investigated the differential expressed genes (DEGs) in each cell type in pairwise comparison between sample classes and specifically expressed in the cell type at the same time. These genes were identified as marker genes for the cell groups (see Method). In total 709 genes were identified to play a vital role in the immune microenvironment of LUAD (Figure 2A). Interestingly, Epithelial cell has 629 marker genes, which reach the most, while the number of epithelial cells is only a small part of the total number of cells (5%). The cell with the second highest number of marker genes was cytotoxic T cell, with 159 marker genes and 7614 cells (20.6%) (Supplementary Table 2).

Investigation of key factors that induce the immune heterogeneity between different sample classes would provide a glimpse of the different malignancies and metastasis of LUAD patients in different pathological conditions. Memory T cell was the prevalent cell in tumor tissue and 26 genes were identified to be significantly differentially expressed between normal and tumor samples (Figure 2B), which were specifically expressed in memory T cells. HSPA1A and HSP90AA1 were the most significant ones. Whereas JUN, HSPH1, HSPA8, HSPA6, HSP90AB1, and EGR1 were found to be the DEGs in both Tumor vs. normal and tumor vs. metastasis group. In addition, M1/M2 macrophages were enriched in normal tissue (Figure 2C). IGKC, SCGB1A1 HSPB1, and FCGR3A were found to be differentially expressed in normal tissue compared with tumor and metastasis. Especially for metastasis tissue, Helper T cells were the most common cells and 119 markers were identified, such as VIM, ZFP36L2, TSC22D3, RPS29, and PRL41. These results reveal the molecular characteristics of different cell subtypes in different sample classes, thus facilitating our understanding of the microenvironment of LUAD.

### Pathways analysis revealed immune cell heterogeneity

**Figure 3:**
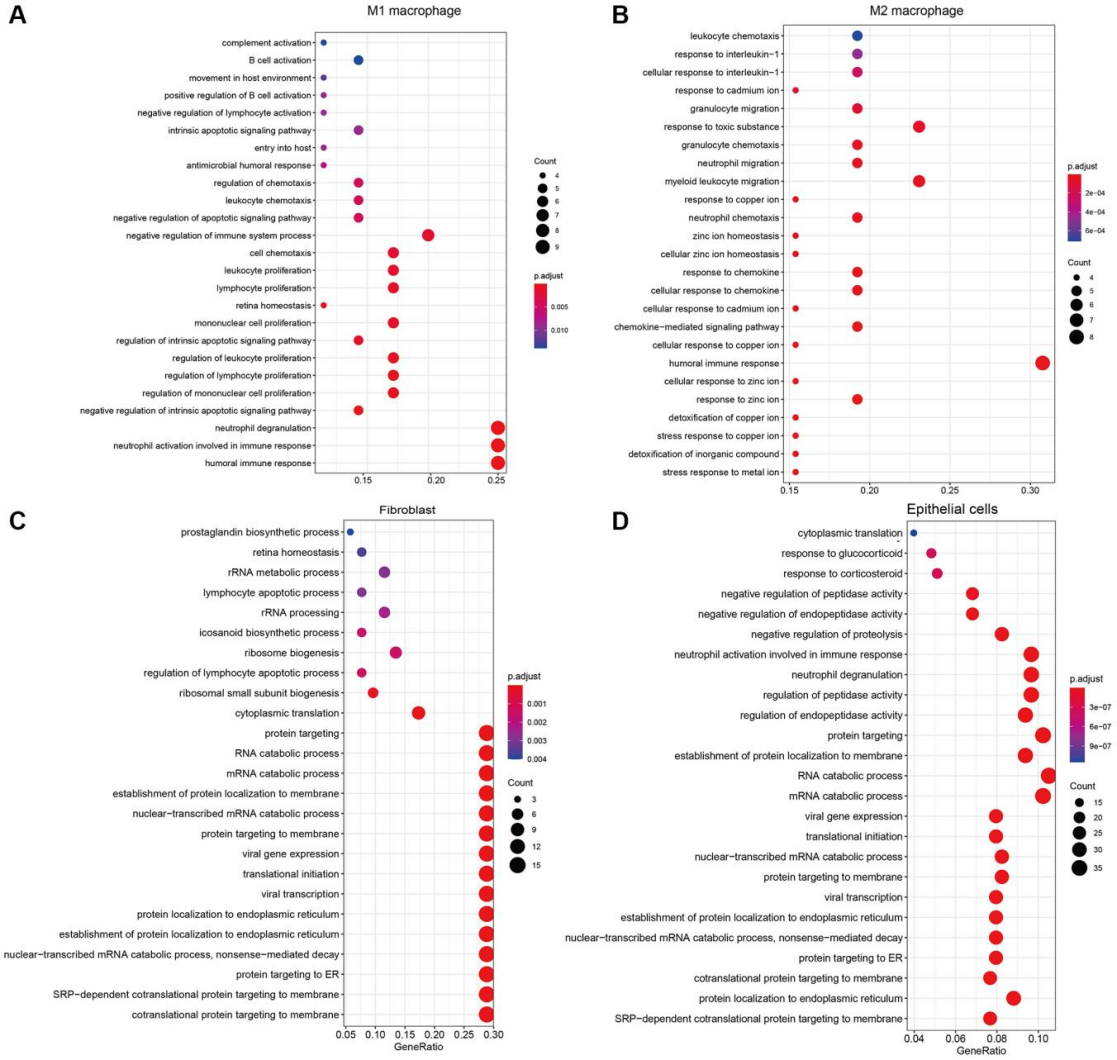
The results of GO enrichment analysis for marker genes of (**A**) M1 macrophages, (**B**) M2 macrophages, (**C**) Fibroblasts, (**D**) Epithelial cells.

Specifically expressed genes contribute significantly to the heterogeneity between different sample classes and we further applied enrichment analysis to the marker genes to explore the biological mechanism affecting the microenvironment of LUAD. The results of GO enrichment analysis suggested that marker genes of M1 macrophage were enriched in immune-related pathways, such as “involved in immune response”, “neutrophil degranulation” and “regulation of mononuclear cell proliferation” (Figure 3A). Besides, markers of M2 macrophage enriched in not only immune-related pathways, such as “cellular response to chemokine”, “neutrophil chemotaxis” and “myeloid leukocyte migration” ion transportation pathways were also emphasized, such as “stress response to meta ion”, “response to zinc ion” and “cellular response to copper ion”(Figure 3B). For non-immune cells, the enrichment results of marker genes show that the cells are mainly related to protein targeting and localization (Figure 3C, 3D). Overall, the enrichment results of marker genes matched the functions of cell types in our cognition and deepened our understanding of the microenvironment of LUAD.

**Figure 4:**
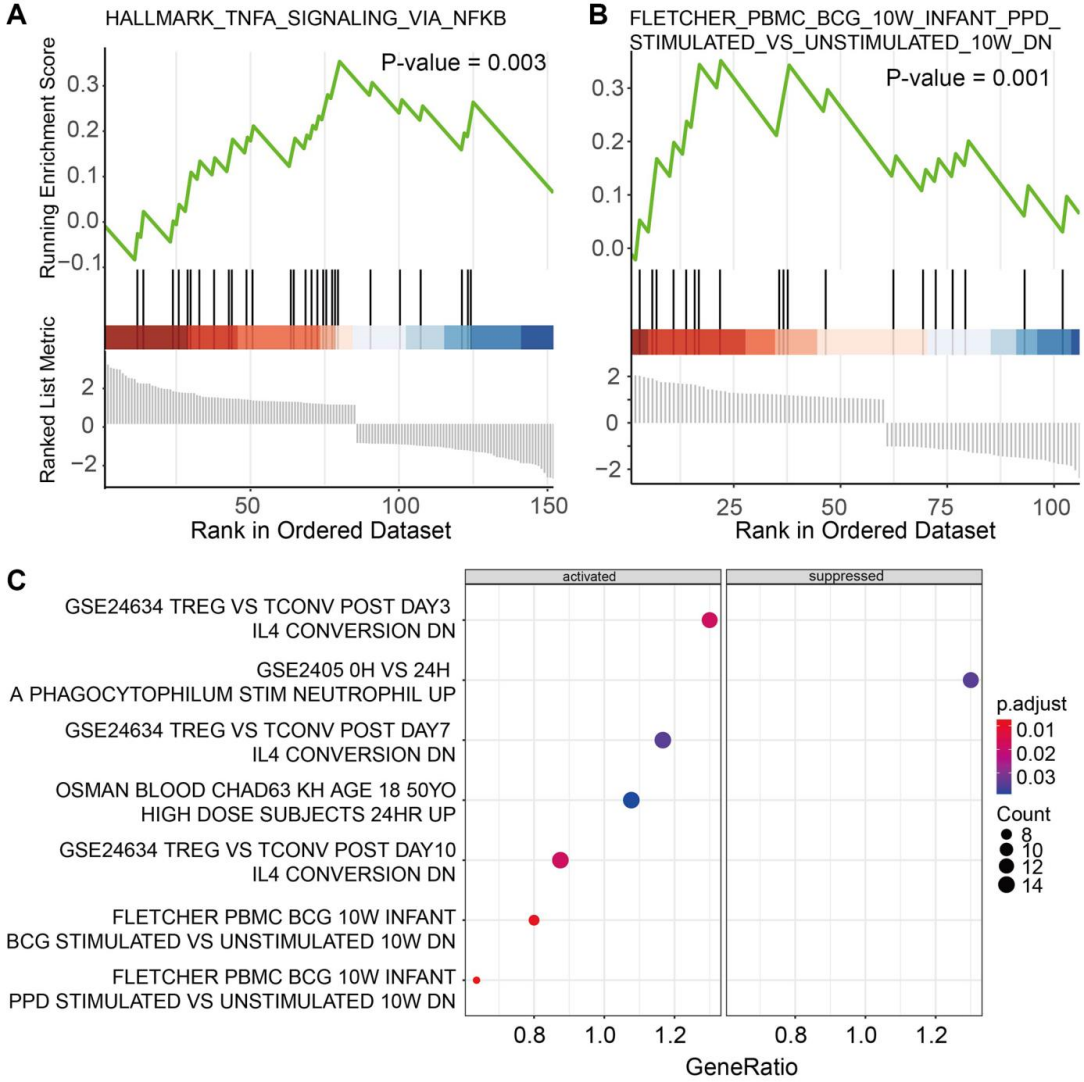
Functional analysis of marker genes for (**A**) cytotoxic T cells, (**B**) Helper T cells, and (**C**) Monocytes.

Functional analysis of marker genes showed that immune cells play an important role in affecting the cancer microenvironment through immune-related pathways. Further GSEA suggested that cytotoxic T cells were significantly enriched in 2 cancer hallmarks and 9 immune signatures from MigDB and indicated to be active in response to tumor necrosis factor (TNF) with the regulation of NFKB (Figure 4A). In addition, Helper T cells were founded to be enriched in genes positively correlated with memory B cell response (Figure 4B). Monocytes were active in the genes regulated by IL4, while suppressing the activity of polymorphonuclear leukocytes (Figure 4C). For non-immune cells. The marker genes of mesenchymal progenitors enriched in cancer hallmarks of epithelial-mesenchymal transition only. These results suggested broad communications between different subtypes of immune cells.

### Cell-cell communication

**Figure 5:**
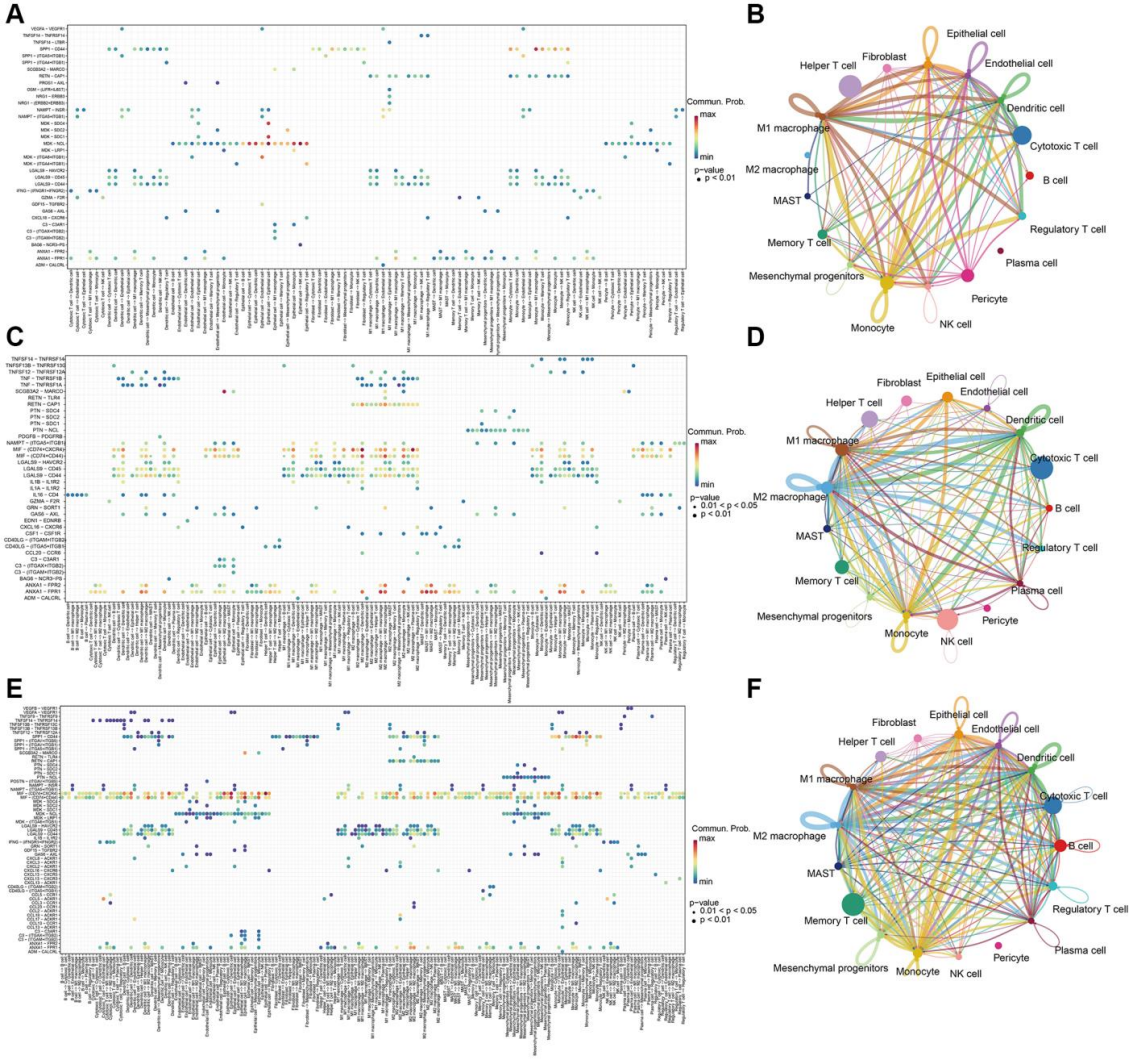
Cell-cell communication analysis with CellChat in (**A**, **B**) metastasis samples, (**C**, **D**) Normal samples, and (**E**, **F**) tumor samples.

The enrichment analysis results indicate that there is a wide range of cell-cell interactions between different types of cells and the tumor microenvironment is characterized by these complex interactions. To interrogate putative cell-cell interaction heterogeneity in different sample classes, we applied CellChat, which includes broad ligand-receptor pairs, to gain insights into interactions among all immune cell types. As a result, pericytes, which are predominant in metastasis tissues, were revealed to have broad interactions with B cells, cytotoxic T cells, dendritic cells, endothelial cells, epithelial cells, M1 macrophage, memory T cells, regulatory T cells, monocytes, and NK cells via the MDK-NCL pathways, which is an important hallmark in the change of tumor microenvironment, and serves as a sign of cancer-associated fibroblasts (CAF) activation to stimulate downstream pathways for facilitating tumor invasion [42]. What’s more, Endothelial cells and epithelial cells were also found to be active in MDK-NCL pathways in communication with immune cells (Figure 5A, 5B). Besides, MIF-(CD74+CXCR4) and MIF-(CD74+CD44) pathways were widespread and are associated with multiple cell types in the tumor and normal tissues. Macrophage migration inhibitory factor (MIF) is an inflammatory cytokine that exhibits chemokine-like activities through non-cognate interactions with the chemokine receptors CXCR2 and CXCR4, in addition to activating the type II receptor CD74. Activation of the MIF-CXCR4 axes promotes leukocyte recruitment, mediating the exacerbating role of MIF in atherosclerosis [43]. MIF-(CD74+CXCR4) and MIF-(CD74+CC44) interactions happened in dendritic cells, M1 macrophage, M2 macrophage, monocyte, and plasma cells both in tumor and normal tissues (Figure 5C, 5D). Except for the same cell type in normal tissue, tumor tissues were also found B cell, cytotoxic T cell, MAST cell, memory T cell, and regulatory T cell to have the interaction (Figure 5E, 5F), which uncovers the change of microenvironment between tumor and normal tissues.

### Regulatory element analysis

**Figure 6:**
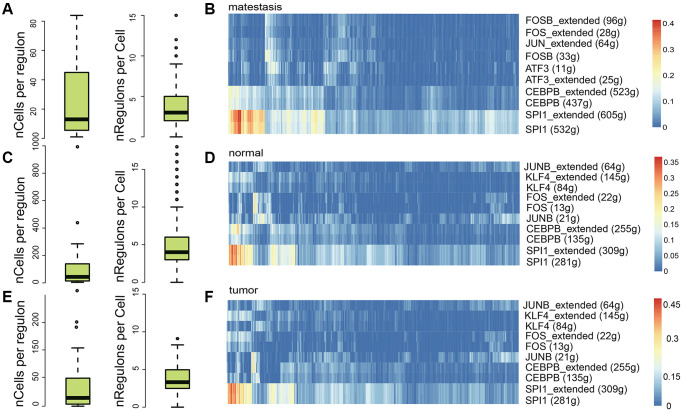
**Regulatory element analysis with SCENIC in different sample classes.** The number of cells per regulon and the regulon activity in metastasis (**A**, **B**), normal (**C**, **D**), and tumor (**E**, **F**) samples, respectively.

To further dissect the immune heterogeneity of gene regulation, the single-cell regulatory network inference and clustering (SCENIC) analysis for DEGs was performed to assess the differences in the expression levels of transcription factors (TFs) between different sample classes. In general, metastasis, normal, and tumor tissues contained a similar number of regulons per cell (RPC), while in normal tissues, the number of cells per regulon (CPR) was significantly higher (Figure 6A, 6C, 6E), which indicate that the microenvironment was more complex in metastasis and tumor tissue. The most five active regulators were identical in tumor and normal tissues, SPI1, CEBPB, JUNB, FOS, and KLF4 (Figure 6B, 6D, 6F). While in metastasis tissue, ATF3 was the third active regulator, which is a stress-induced transcription factor that plays a vital role in modulating metabolism, immunity, and oncogenesis [44].

### Immune cell abundance prediction

**Figure 7:**
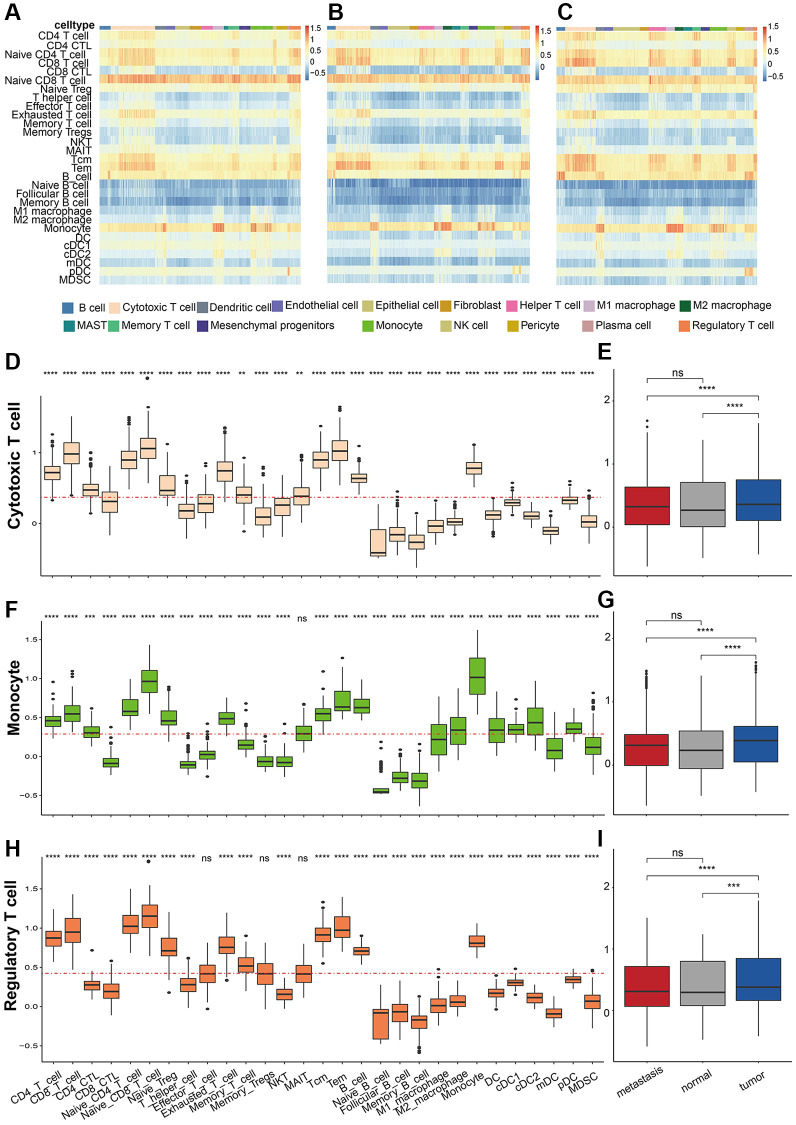
**Immune cell abundance prediction.** Heatmap of Estimate score in (**A**) metastasis, (**B**) normal samples, and (**C**) tumor samples in different cells. The predicted estimate score of cytotoxic T cell markers in (**D**) different cells and (**E**) samples. The predicted estimate score of monocyte markers in (**F**) different cells and (**G**) samples. The predicted estimate score of regulatory T cell markers in (**H**) different cells and (**I**) samples. *P* values were shown as: ^*^p < 0.05, ^**^p < 0.01, and ^***^p < 0.001.

To further unveil the role of the immune cells in their microenvironment in different sample classes, we then investigated the immune cell type composition through ssGSEA algorithm (see Method). As a result, the marker genes of Naïve CD4 T cell, Naïve CD8 T cell, exhausted T cell, T central memory (Tcm), T effector memory (Tem), B cell, and monocyte had relatively high enrichment fraction in all cells (Figure 7A–7C). The proportion of cytotoxic T cells in each sample was similar, but the analysis of immune cell abundance showed that the enrichment fraction of CD4+ T cells with cytotoxic activity was significantly higher in cytotoxic T cells than in other immune cells, like regulatory T cells and memory T cells (Figure 7D). Besides, the enrichment scores of all the immune cells in Cytotoxic T cells were significantly higher in tumor tissues than in metastasis and normal tissue (Figure 7E). Unsurprisingly, monocytes had higher enrichment scores for marker genes in monocyte, M1 macrophage, and M2 macrophage (Figure 7F). Besides, monocytes also had significantly high enrichment scores in type 2 conventional dendritic cells, while the scores of memory dendritic cells in monocyte were significantly low (Figure 7G–7I). These results indicated that monocyte may exhibit vital biological functions in LUAD.

### Marker gene validation in bulk RNA-seq

**Figure 8:**
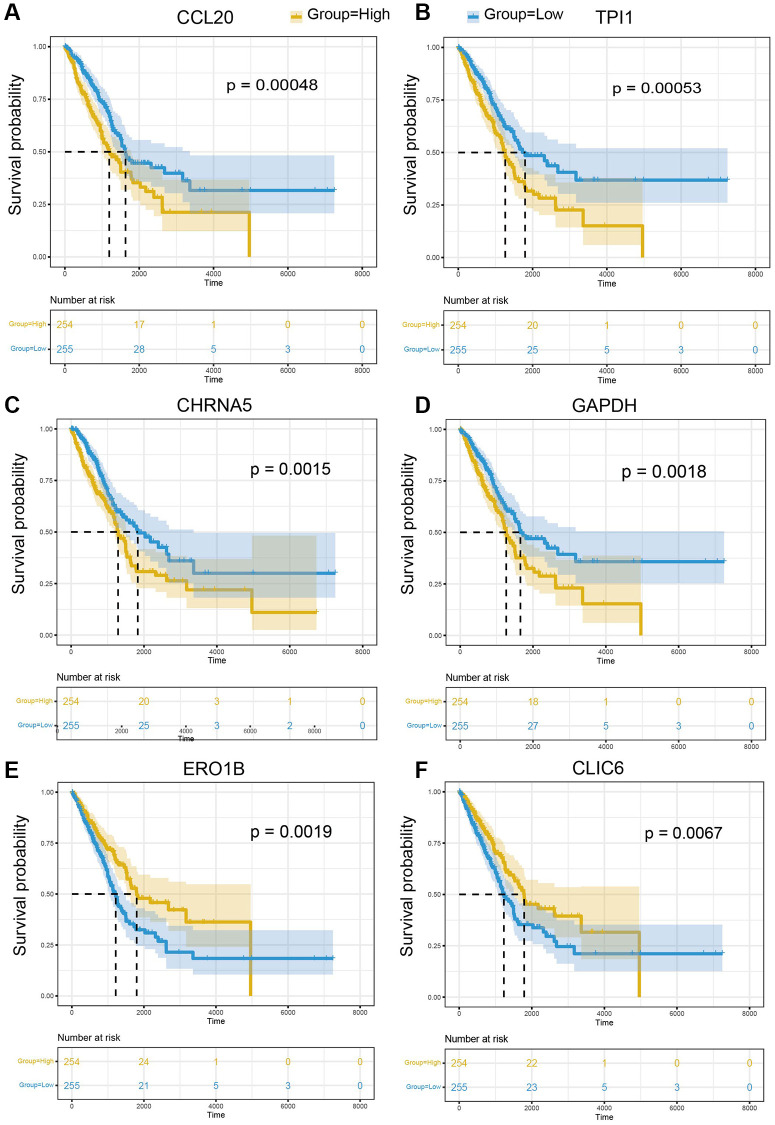
**The prognostic significance of marker genes in LUAD.** Kaplan-Meier survival curves of (**A**) CCL20, (**B**) TPI1, (**C**) CHRNA5, (**D**) GAPDH, (**E**) ERO1B, (**F**) CLIC6 in LUAD. The median of gene expression is utilized as the cut-off. Yellow: higher expression of indicated gene; Blue: lower expression of indicated gene.

To further explore the impact of cell-type specifically expressed marker genes on LUAD prognosis, mRNA expression matric and clinical information of TCGA-LUAD were collected and applied to survival analysis. The results of survival analysis suggested that CCL20, a marker of M2 macrophage, is the most significant gene related to LUAD prognosis (*p*-value = 0.00048) (Figure 8A) and patients with low expression of CCL20 have a better prognosis. Besides, TPI1 is the marker gene of Pericyte and patients with low expression of TPI1 have a better prognosis (*p*-value = 0.00053) (Figure 8B). Epithelial cells have 16 markers genes significantly correlated with LUAD prognosis, including CHRNAA5 (*p*-value = 0.0015) (Figure 8C), GAPDH (*p*-value = 0.0018) (Figure 8D), ERO1B (*p*-value = 0.0019) (Figure 8E), and CLIC6 (*p*-value = 0.0067) (Figure 8F). In general, 25 marker genes of 10 cell types were identified to be significantly related to LUAD prognosis and these genes provide molecular insight into the function of specific cell types in LUAD pathology (Supplementary Table 3).

## DISCUSSION

In this study, we collected normal, tumor, and metastasis LUAD datasets in single-cell sequencing and applied bioinformatic methods to explore the distinct molecular features of different sample types. As a result, the cellular landscapes of different subclasses of LUAD were depicted. With the bioinformatic method (see Method), marker genes of each cell type in LUAD were identified and enrichment analysis was used to explore the biological function of these marker genes. Combined with trajectory analysis and cell-cell communication analysis, the different microenvironments between different sample classes were characterized. For example, CCL20 encodes protein displaying chemotactic activity for lymphocytes and can repress the proliferation of myeloid progenitors. Previous research reported that high CCL20 expression is a poor prognostic marker for LUAD patients, and is associated with enhanced epithelial-mesenchymal transition (EMT), inflammatory response, and activated TNF pathway in LUAD. While low CCL20 expression blocked the detrimental effects of high TGF-β on survival and effectively improved patients’ response to anti-PD-L1 therapy [45]. In our study, CCL20 is specifically expressed in M2 macrophage, and the expression in normal samples is much higher than in other samples, which indicates that M2 macrophage plays an important role in LUAD through the function of CCL20. Further enrichment analysis of marker genes of LUAD revealed that the significantly enriched GO terms were chemokine-mediated signaling pathway, myeloid leukocyte migration, positive regulation of leukocyte migration, calcium-mediated signaling, and positive regulation of ERK1/ERK2 cascade. Besides, IL-17 signaling pathway and TNF signaling pathway were the most significant KEGG pathways. Marker genes shared in these pathways were TREM2, CXCL3, CCL20, IL1B, CXCL8, and CCL4. Then, the result of cell-cell communication analysis illustrated different signal pathways in different sample classes. In normal samples, CCL20-CCR6 interaction occurs in M2 macrophage->DC, M2->Treg. The ligand-receptor pair CCL20-CCR6 is responsible for the chemotaxis of dendritic cells (DC), effector/memory T-cells, and B-cells, which plays an important role at skin and mucosal surfaces under homeostatic and inflammatory conditions, as well as in pathology, including cancer and various autoimmune diseases. While in meta samples, macrophage function is weaker. In conclusion, this study has revealed the characteristic of normal, tumor, and metastasis LUAD tissues and associated immune microenvironment, and further illuminated changes in cellular and molecular dynamics in different sample classes.

## Supplementary Materials

Supplementary Table 1

Supplementary Table 2

Supplementary Table 3
