# Supplementary material for: Heterogeneity of tumor immune microenvironment in malignant and metastatic change in LUAD is revealed by single-cell RNA sequencing
Source: Aging (Albany NY). 2023 Jun 16;15(12):5339–54. doi: 10.18632/aging.204752 (PMC10333068; doi:10.18632/aging.204752)
Supplement: Supplementary Table 1 [file aging-15-204752-s001.pdf]

SUPPLEMENTARY TABLE

Supplementary Table 1. Marker gene list.

Cell type	Marker genes
B cell	CD19, MS4A1, CD79A, CD79B, SPIB
Cytotoxic T cell	CD3D, CD3G, CD8A, CD8B
Dendritic cell	XCR1, CLEC9A, THBD, CD1C, CD1E, FCER1A
Endothelial cell	CDH5, PECAM1, VWF
Epithelial cell	EPCAM, CDH1
Fibroblast	LUM, DCN, COL1A2, COL1A1
Helper T cell	CD3D, CD3G, CD4, TBX21, IFNG, IL4, CXCR5
M1 macrophage	CD68, IL1B, IL6, TNF
M2 macrophage	MRC1, CD163, TGFB1, IL10, FN1
MAST	TPSAB1, TPSB2, CPA3
Memory T cell	PTPRC, CCR7, SELL
Mesenchymal progenitors	FDGFRA, LGR5
Monocyte	CD14, FCN1, FCGR3A, VCAN
NK cell	KLRD1, NKG7, KLRC1, FCGR3A, CD3D-, CD3G-
Pericyte	RGS5, ACTA2, MCAM, PDGFRB, NOTCH3
Plasma cell	CD79A, JCHAIN, MZB1, IGHG1
Regulatory T cell	FOXP3, IL2RA, CTLA4, IKZF2, CD25
